# The serum level of C-reactive protein (CRP) is associated with cognitive performance in acute phase psychosis

**DOI:** 10.1186/s12888-016-0769-x

**Published:** 2016-03-14

**Authors:** Erik Johnsen, Farivar Fathian, Rune A. Kroken, Vidar M. Steen, Hugo A. Jørgensen, Rolf Gjestad, Else-Marie Løberg

**Affiliations:** Division of Psychiatry, Haukeland University Hospital, Bergen, Norway; Department of Clinical Medicine, Section Psychiatry, University of Bergen, Bergen, Norway; NKS Olaviken Gerontopsychiatric Hospital, Bergen, Norway; NORMENT and KG Jebsen Centre for Psychosis Research, Department of Clinical Science, University of Bergen, Bergen, Norway; Dr. Einar Martens Research Group for Biological Psychiatry, Center for Medical Genetics and Molecular Medicine, Haukeland University Hospital, Bergen, Norway; Department of Clinical Psychology, University of Bergen, Bergen, Norway; Department of Addiction Medicine, Haukeland University Hospital, Bergen, Norway

**Keywords:** Schizophrenia, Cognition, Inflammation, CRP

## Abstract

**Background:**

Inflammatory processes have been implicated in the etiology of schizophrenia and related psychoses, in which cognitive deficits represent core symptoms. The aim of the present study was to investigate possible associations between the level of the inflammation marker C-reactive protein (CRP) and cognitive performance in patients through the acute phase of psychosis.

**Methods:**

A total of 124 patients were assessed at admittance to hospital and 62 patients were retested at discharge or after 6 weeks at the latest, with measurements of the CRP levels and alternative forms of the Repeatable Battery for the Assessment of Neuropsychological Status.

**Results:**

There was an inverse relationship between overall cognitive performance and CRP level at admittance. The association increased in sub-analyses including only patients with schizophrenia. In cognitive subdomain analyses statistically significant inverse associations were found between the CRP level and Delayed memory and Attention, respectively. No associations were found between CRP level and other measures of psychopathology including psychosis symptoms, depression, or functioning. At follow-up the association between CRP level and cognition was no longer present. There was a significant increase in cognitive performance between baseline and follow-up. There was a stronger increase in overall cognition scores in patients with higher baseline CRP levels.

**Conclusions:**

The findings indicate that signs of inflammation may serve as a state-dependent marker of cognitive dysfunctions in acute psychosis.

**Trial registration:**

ClinicalTrials.gov ID; NCT00932529, registration date: 02.07.2009

## Background

Schizophrenia and related psychoses are severe mental disorders characterized by positive and negative psychotic symptoms, cognitive dysfunction and functional decline, with a lifetime prevalence close to 1 % [1]. Positive symptoms were until recently considered the most prominent features of the disorders as reflected also in the major diagnostic manuals, but cognitive dysfunctions have for the last decade been recognized as core features of schizophrenia [2–5], and with greater impact on functional outcome than the psychotic symptoms [6–10]. The etiology of schizophrenia remains to be clarified, but genetic as well as environmental factors convey risk [11]. Involvement of inflammation and the immune system in the pathophysiology of schizophrenia has received particular attention in recent years, fueled also by the genome wide association study (GWAS) findings of associations between markers in the immune system including the major histocompatibility complex and schizophrenia risk [12–15]. Furthermore, preclinical-, postmortem-, brain imaging-, and pharmacological studies, as well as clinical evidence from drug naïve first episode patients, strongly suggest a role for the immune system in schizophrenia development (see for example [16–18] for updated reviews). Emerging evidence indicates that immuno-inflammatory processes may be particularly relevant to the cognitive dysfunctions of schizophrenia [19–21].

The C-reactive protein (CRP), an acute-phase reactant synthesized in the liver, has for many decades been considered a reliable marker of inflammation [22]. With regards to cognition, a negative correlation has been found between CRP levels and cognitive impairment in the elderly, although not consistently [23, 24]. Scattered reports also exist of inverse associations between CRP levels and cognitive function in severe depression [25], and in bipolar disorder [26]. In schizophrenia, a recent meta-analysis by Fernandes et al. [27] consistently found elevated serum levels of CRP in both first episode and chronic phase patients, irrespective of medication status. Furthermore, an association between CRP levels and positive symptoms but not negative symptoms of psychosis was found. To the best of our knowledge, investigations of associations between the CRP level and cognitive dysfunction in schizophrenia are however scarce, although an association between CRP levels and cognitive functioning in patients with predominantly chronic schizophrenia has been reported in one cross-sectional study [28]. Studies involving patient samples representative of the acute and early phases of psychosis are missing, as are studies with longitudinal measurements. We have previously demonstrated a statistically significant time effect for overall cognitive improvement in acutely admitted psychosis patients during 24 months of follow-up [29], but have not so far examined changes in the acute phase.

The main aim of our study was accordingly to investigate the association between the CRP level and cognitive performance in a clinically representative sample of patients with psychosis acutely admitted to hospital, with repeated measurements in the acute phase.

## Methods

The materials and methods used have been described in greater detail elsewhere [30]. The study is part of a pragmatic, randomized trial comparing second generation antipsychotics (SGAs) in the treatment of psychosis. The present paper reports data obtained at baseline in patients who underwent cognitive assessments at admittance and at discharge or after maximally 6 weeks if not already discharged (termed as follow-up). This time period corresponds to the acute phase of treatment. Patients were consecutively recruited from March 2004 until February 2009 from the Haukeland University Hospital with a catchment population of about 400,000. The study was approved by the Regional Committee for Medical Research Ethics, and by the Norwegian Social Science Data Services. The study was publicly funded and did not receive any financial or other support from the pharmaceutical industry. The Regional Committee for Medical Research Ethics allowed eligible patients to be included before informed consent was provided, thus entailing a clinically relevant representation in the study. The patients were asked at follow-up for written informed consent. All adult patients were eligible for the study if they were acutely admitted to the emergency ward for symptoms of active psychosis as determined by a score of ≥4 on one or more on the items Delusions, Hallucinatory behavior, Grandiosity, Suspiciousness/persecution, or Unusual thought content in the Positive and Negative Syndrome Scale (PANSS) [31] and were candidates for oral antipsychotic drug therapy. Accordingly the patient inclusion encompassed the consecutive recruitment of a clinically representative sample of psychosis patients acutely admitted to hospital. All eligible patients met the ICD-10 diagnostic criteria (http://apps.who.int/classifications/icd10/browse/2010/en) for schizophrenia, schizoaffective disorder, acute and transient psychotic disorder, delusional disorder, drug-induced psychosis, bipolar disorder except manic psychosis, or major depressive disorder with psychotic features. The diagnoses were determined by the hospital’s psychiatrists or specialists in clinical psychology. Patients were excluded from the study if they were unable to use oral antipsychotics, were suffering from manic psychosis or for other behavioural or mental reasons related to the state of illness were unable to cooperate with assessments, did not understand spoken Norwegian, were candidates for electroconvulsive therapy, or were medicated with clozapine on admittance. Patients with drug-induced psychoses were included only when the condition did not resolve within a few days and when antipsychotic drug therapy was indicated.

### Clinical assessments

Before inclusion, eligible patients underwent the PANSS structured clinical interview. Intra-class correlation coefficients (ICC) were calculated based on inter-rater assessments and showed high inter-rater reliability (0.92). Furthermore, the Calgary Depression Scale for Schizophrenia (CDSS) [32], and the Clinical Drug and Alcohol Use Scales (CDUS/CAUS) [33] were used, and the patients were rated according to the Clinical Global Impression—Severity of Illness scale (CGI-S) [34], and the Global Assessment of Functioning—Split Version, Functions scale (GAF-F) [35]. A blood sample was collected from the patients between 08 and 10 a.m. for analyses of CRP levels. There was a change in the laboratory’s CRP analysis methods in January 2005, and hence only data obtained after this change is reported in the present work. The method used is the Tina-quant C-reactive Protein (Latex) from Roche Modular P®, which measures CRP levels >1 mg/L. Antipsychotic drug doses were converted to defined daily doses (DDDs), in accordance with the World Health Organization Collaborating Center for Drug Statistics Methodology (http://www.whocc.no/atc_ddd_index/). The basic definition of the DDD unit is the assumed average maintenance dose per day for a drug used for its main indication in adults.

### Cognitive assessment

Cognitive assessments were conducted at baseline and at follow-up. A brief neuropsychological screening instrument with alternative forms; the Repeatable Battery for the Assessment of Neuropsychological Status (RBANS), was used to minimize potential practice effects [36–38], since longitudinal studies on cognitive functioning usually do not address the issue of practice effects sufficiently [39, 40]. Practice effects can be particularly evident when there are short time intervals between repeated neuropsychological testing, and the effect seems to be strongest from baseline to the second testing [41, 42]. The RBANS has previously shown good reliability and validity in psychosis [43]. It takes only about 30 min to complete, making it practical and feasible to use in the acute phases of psychosis. The five cognitive domains were: Language; Visuospatial/ constructional; Immediate memory; Delayed memory; and Attention. Raw scores from the neuropsychological variables were converted to t-scores by means of the norms from the manual [44]. The final summary score based on the mean t-scores across the five cognitive domains defined the overall cognitive function t-score.

### Statistical procedures

Categorical and continuous data at baseline were analyzed using exact *χ*^2^ – tests and one-way ANOVAs in the SPSS software, version 20.0 (IBM SPSS Statistics, 2011). To investigate the association between cognitive performance and CRP levels bivariate analyses of correlation were conducted. This was followed by linear regression analyses to adjust for potential confounders between cognition and CRP. These confounders included years of education, as a proxy for socioeconomic status which may have an impact on both CRP levels and cognitive performance [45]; medication status (i.e. being antipsychotic drug naïve or previously exposed to antipsychotic drugs) prior to inclusion, as antipsychotics may influence both CRP levels and cognition [46, 47]; tobacco smoking, which has been associated with both elevation of CRP levels [48, 49] and enhancement of cognition [50, 51]; drug abuse, as a relationship between drug abuse and CRP has been established [52, 53]; and finally, cardiovascular risk, as CRP has been identified as a risk factor for cardiovascular disease (CVD) [54, 55] and CVD has been associated with cognitive impairment [56, 57]. A CVD risk score was calculated based on the International Diabetes Federation metabolic syndrome definition cut-off values (http://www.idf.org/webdata/docs/IDF_Meta_def_final.pdf), by which each factor (obesity, raised triglycerides, reduced HDL cholesterol, raised blood pressure, and raised fasting plasma glucose) was dichotomized as absent (0) or present (1), giving rise to a maximal sum score of 5 for the individual factors.

Latent Growth Curve (LGC) models of level and change in CRP and cognition were analyzed with the Mplus program, version 7.20 [58]. Such models describe both mean levels and individual variations in level and change. In addition, the relation between level and change is estimated. Because of only two measurement points, baseline and follow-up, the residuals had to be pre-specified in order to identify the model [59]. Mplus allows unequal individual time-spaced observations to be analyzed [60], and time was specified as weeks. The default estimator for LGC modelling is maximum likelihood with robust standard errors (MLR), which is robust for non-normal data [60, 61]. Standard Mplus models use all available data under the “missing at random” assumption and minimize the effect of missing data [62, 63]. First, unconditional separate LGC models were analyzed, then a model integrating level and change in both CRP and cognition was used in order to study the relation between baseline levels in one variable as a predictor of changes in the opposite variable, after accounting for the control covariates. Variables not accounting for any relations were removed and model re-estimated based on a backward hierarchical procedure [64].

The level of statistical significance was set at α = 0.05, two-sided.

## Results

A total of 124 patients were included with serum CRP level measurements and cognitive assessments at baseline. The demographic and clinical characteristics are shown in Table 1. One patient used concomitant anti-inflammatory medication (prednisolone). None of the included patients were diagnosed with inflammatory- or immunological disorders or infections during the study.

**Table 1:** Baseline demographics and clinical characteristics (*N* = 124)

Characteristics		
Gender	N	% of sample
Male	84	67.7
Female	40	32.3
Antipsychotic drug naïve	64	51.6
Alcohol use last 6 months		
None	17	13.7
Misuse	15	12.1
Illicit drug use last 6 months		
None	82	66.1
Use/ Misuse	25	20.2
Current tobacco smoking	64	51.6
Diagnosis^a^		
Schz and related	67	57.8
Acute	8	6.9
Drug-induced	21	18.1
Affective	11	9.5
Rest	9	7.8
	Mean	SD/ range
Age	33.5	12.4/18–65
Body Mass Index	23.5	4.6/ 15.8–40.3
Years of Education	12.5	2.7/ 8–22
PANSS Total	73.4	11.9/ 45–98
PANSS Positive	20.1	4.1/ 12–30
PANSS Negative	19.0	6.9/ 7–38
PANSS General	34.3	6.4/ 20–56
CDSS	6.2	5.0/ 0–23
GAF-F	30.5	4.9/ 18–45
CGI	5.2	0.6/ 4–6
RBANS t-score	37.8	7.7/ 20.2–58.8

*N* number of patients, *SD* standard deviation; Antipsychotic drug naive = No life-time exposure to antipsychotic drugs before index admission; Misuse = Misuse or Dependence according to the Clinical Drug and Alcohol Use Scales (CDUS/CAUS), patients with no illicit drug use could be included in the category alcohol use last 6 months; Schz and related = Schizophrenia and related disorders: Schizophrenia, schizo-affective disorder, acute polymorphic psychotic disorder with symptoms of schizophrenia, acute schizophrenia-like psychotic disorder, delusional disorder; Acute = Acute psychosis other than those categorized under Schz and related; Affective = Affective psychosis; Rest = Miscellaneous psychotic disorders. All diagnoses are according to ICD-10; *PANSS* the Positive and Negative Syndrome Scale, *CDSS* the Calgary Depression Scale for Schizophrenia, *GAF-F* the Global Assessment of Functioning, split version, Functions scale, *CGI* the Clinical Global Impression, severity of illness scale, *RBANS* the Repeatable Battery for the Assessment of Neuropsychological Status

^a^Patients with missing diagnoses are not included in the list

### Baseline

The mean CRP level with standard deviation (SD) at admission was 3.6 (5.2) mg/L and the mean overall cognitive function t-score was 37.8 (7.7).

**Fig. 1:**
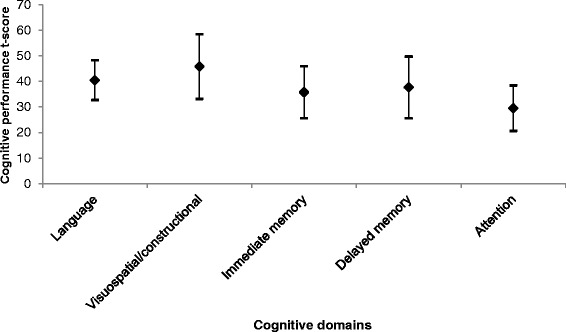
Cognitive performance by functional domain. Notes: ♦ = mean t-score with bars representing plus/minus 1 standard deviation from the mean

The cognitive subdomain t-scores were 40.5 (7.8); 45.8 (12.7); 35.7 (10.2); 37.6 (12.1); and 29.5 (8.9) for Language; Visuospatial/ constructional abilities, Immediate memory; Delayed memory; and Attention, respectively (Fig. 1).

**Fig. 2:**
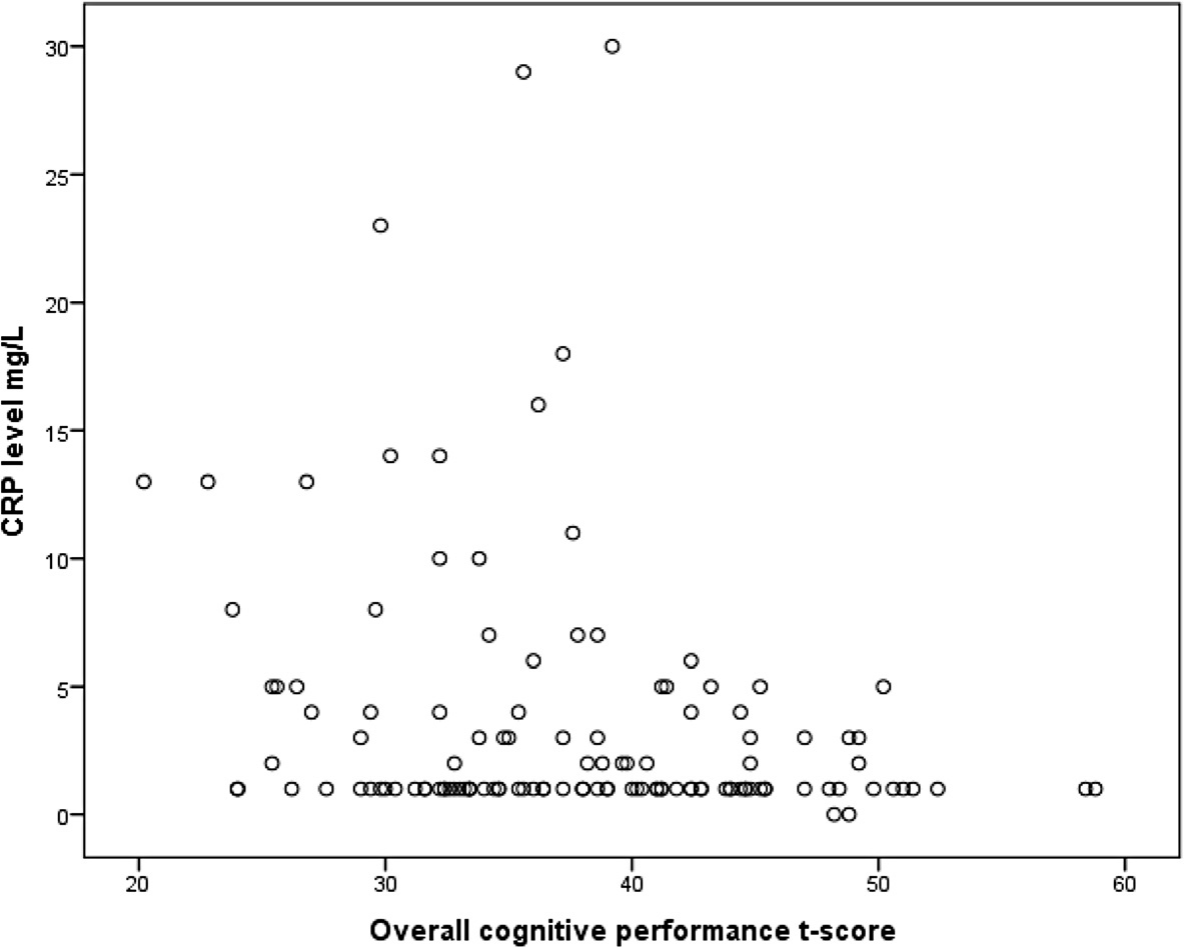
Association between CRP level and overall cognitive performance at baseline. Notes: Scatter plot of overall cognitive performance versus CRP level (*N* = 124). Pearson correlation *r* = −0.247, r2 = 0.061, *p* = 0.006. In sensitivity analyses excluding outliers with CRP >15 (*N* = 119): Pearson correlation *r* = −0.346, r2 = 0.120, *p* = 0.000

In the primary analyses, the Pearson correlation test revealed a statistically significant inverse relationship between overall cognitive performance and CRP level at baseline (Pearson correlation *r* = −0.247, R2 = 0.061, *p* = 0.006) (Fig. 2). In a linear regression model with overall cognitive performance as the dependent variable and CRP, years of education, antipsychotic drug status before inclusion, tobacco smoking status, drug abuse, and CVD risk score as independent variables, the association remained statistically significant between cognitive performance and CRP (*B* = −0.290; Beta = −0.198; *p* = 0.031). No interaction effects were found between CRP and any of the other independent variables that significantly improved the model. In the secondary analyses there were statistically significant inverse associations between CRP level and Delayed memory (*B* = −0.484; Beta = −0.213; *p* = 0.02) and Attention (*B* = −0.404; Beta = −0.239; *p* = 0.012), whereas no association was found between CRP and Language, Visuospatial/constructional abilities, or Immediate memory, respectively. In a sensitivity analysis that included also the PANSS positive symptoms scale score, the results remained unchanged.

In Pearson correlation tests of the relationships between CRP and the PANSS total score, the scores of the PANSS positive, negative, and general psychopathology subscales, the CDSS, the GAF-F, and the CGI, respectively, no statistically significant correlations were found (*r* < 0.100 for all).

Based on visual inspection of the CRP levels versus overall cognitive performance scatterplot, the bulk of data were in the lower end of the CRP levels (Fig. 2). Accordingly sub-analyses were conducted that included only patients with CRP levels <15 mg/ L (*N* = 119). The inverse association between CRP levels and overall cognitive performance was strengthened (*B* = −0.741; Beta = −0.300, *p* = 0.001). In the cognitive subdomains statistically significant associations with CRP levels were found for all domains except Immediate memory. When only the schizophrenia subgroup (*N* = 36) was included, the inverse association between CRP levels and overall cognitive performance was markedly increased ((*B* = −1.031; Beta = -0.529; *p* = 0.006).

In sensitivity analyses that included only the sub-group of drug naïve patients (*N* = 64), the inverse relationship between CRP level and overall cognitive performance remained essentially unchanged compared to in the primary analyses with the full sample, although the correlation was no longer statistically significant (*B* = −0.568; Beta = −0.194; *p* = 0.187).

In sensitivity analyses excluding the patient using prednisolone the results were unchanged.

### Follow-up

A total of 62 patients were retested using the RBANS B at follow-up. The mean interval between baseline and follow-up was 28.3 (11.1) days. The mean PANSS total and CGI-S scores were 53.9 (13.9) and 3.6 (1.0), respectively, corresponding to being mildly ill. There were no statistically significant differences between those tested only at baseline and those with follow-up tests for any of the clinical or demographic characteristics at baseline presented in Table 1. Medication details are displayed in Table 2.

**Table 2:** Antipsychotic drug use at discharge/ 6 weeks

	Risperidone	Olanzapine	Quetiapine	Ziprasidone	Aripiprazole
	*N* = 14	*N* = 21	*N* = 14	*N* = 10	*N* = 1
	Mean (SD/ range)	Mean (SD/ range)	Mean (SD/ range)	Mean (SD/ range)	Mean (SD/ range)
Mean dose (mg/ d)	3.3 (1.2/2.0–6.0)	16.6 (4.7/10.0–25.0)	480.4 (218.9/175.0–800.0)	82.0 (38.2/20.0–160.0)	5.0 (-)
Serum level (nm/ L)^a^	58.5 (33.3/ 27.0–147.0)	115.5 (70.3/47.0–302.0)	546.8 (585.3/ 62.0–1817.0)	88.6 (89.3/ 13.0–323.0)	141 (-)

*N* number of patients, *SD* standard deviation, *mg/d* milligrams per day, *nm/L* nanomoles per litre. There was missing medication data on 1 patient and 1 patient had discontinued the antipsychotic medication

^a^Reference ranges: Risperidone 30–120; Olanzapine 30–200; Quetiapine 100–800; Ziprasidone 30–200; Aripiprazole 200–1300

**Fig. 3:**
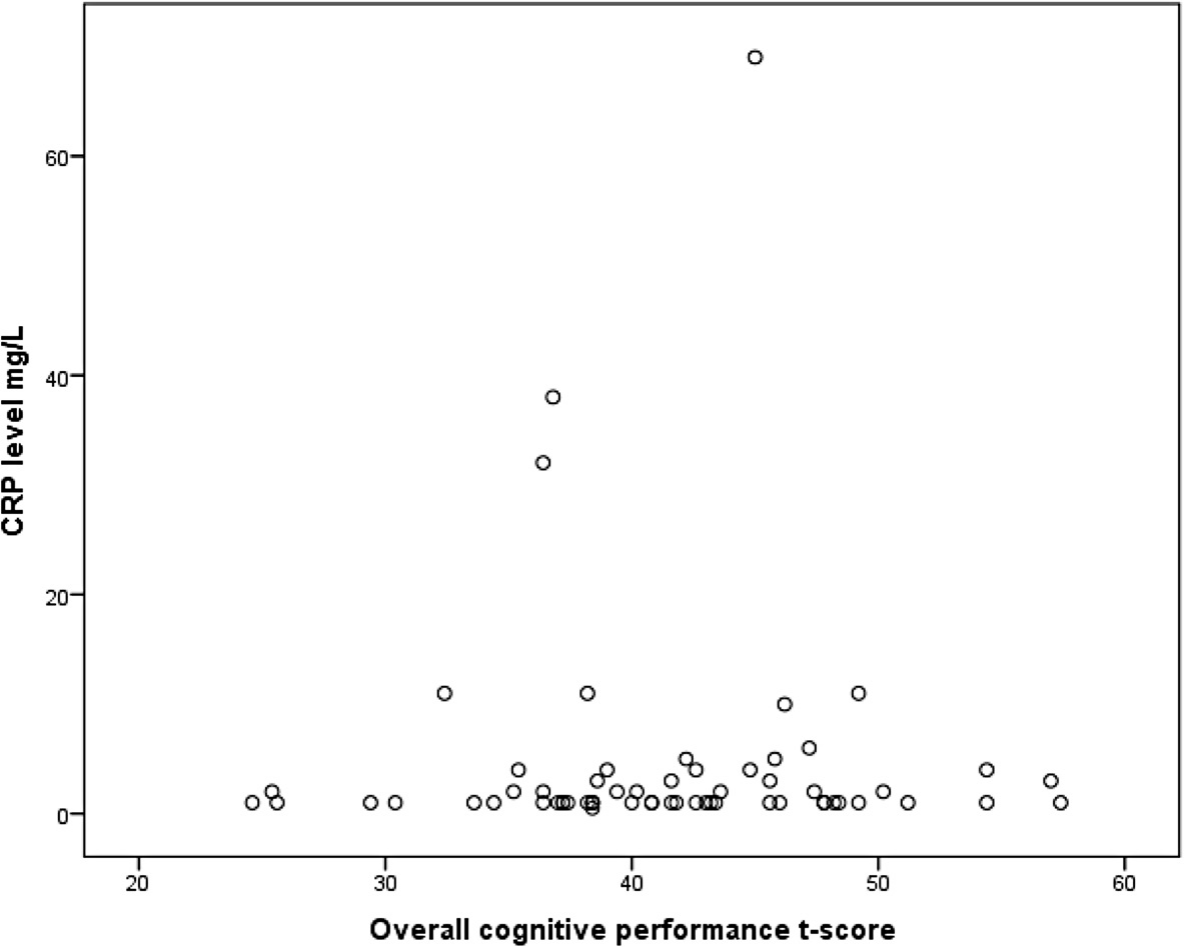
Association between CRP level and overall cognitive performance at follow-up. Notes: Scatter plot of overall cognitive performance versus CRP level (*N* = 62). Pearson correlation *r* = 0.007, r2 = 0.000, *p* = 0.958. In sensitivity analyses excluding outliers with CRP >15 (*N* = 60): Pearson correlation *r* = 0.005, r2 = 0.000, *p* = 0.972

The distribution of CRP levels and overall cognitive performance is displayed in Fig. 3. The mean CRP level and overall cognitive performance were 4.6 (10.6) mg/L and 41.3 (7.1), respectively. The association between CRP level and overall cognitive performance was not statistically significant at follow-up (*B* = −0.045; Beta = −0.066; *p* = 0.627). In sensitivity analyses that included also duration of treatment between baseline and follow-up, as well as the mean defined daily dose of antipsychotics for the latter 7 days before follow-up, the results remained unchanged. In sensitivity analyses excluding the single patient using prednisolone, the results were also unchanged.

The results from the LGC models are presented in Table 3 with mean and individual variation in baseline level and change, and the relation between baseline level and change. There was no statistically significant mean increase in CRP, but considerable and statistically significant individual variation in the change (Table 3). The relation between baseline level and change was not statistically significant.

**Table 3:** Level and change results for CRP level and overall cognition based on latent growth curve models

	Baseline (I)		Change (S)		Relation _I,S_	
	Mean	Variance	Mean	Variance	Cov	r
CRP level	2.76***	9.70	0.98	26.52	−1.33	−.08
Overall cognition	37.92***	60.83	0.95***	3.66	−5.53	−.37**

The model describes mean level and individual variations in baseline and change over time. The relation between intercept (I) and slope (S) describes the relation between baseline level and rate of change (covariances and correlations). *Cov* covariance, *r* correlation coefficient

** *P* < .01, *** *P* < .001

**Fig. 4:**
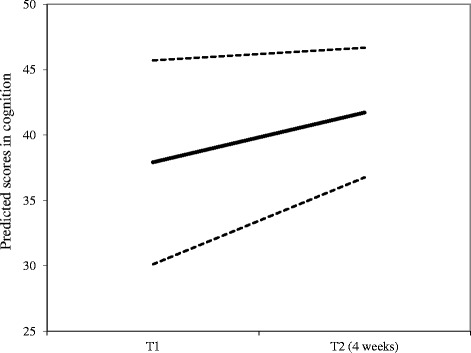
Model estimated cognitive change during follow-up. Notes: The figure shows mean change and change based on ± 1 SD baseline level. Patients with lower cognitive scores had a higher rate of change. SD = standard deviation

Regarding cognition, there was a statistically significant mean increase over time, however; with some patients changing more than others indicated by statistically significant variance. For this variable it was found a negative relationship between baseline score and degree of change, which indicates stronger rate of change for patients with lower baseline scores. Figure 4 illustrates this with mean change over a four week period and changes for patients with lower (−1 SD) and higher (+1 SD) baseline scores.

The results from the final model consisting of two growth processes with level and change in CRP and overall cognition showed that baseline level in CRP could predict changes in overall cognition (*b* = 0.04, *p* = 0.000), after accounting for the covariate variables. Changes in overall cognition were not related to changes in CRP (*b* = −0.02, *p* = 0.764). The covariate variables showed smokers to have less increase over time than non-smokers (*b* = −0.18, *p* = 0.001) and that baseline CRP level was moderated by the CVD risk baseline level (interaction term: *b* = −0.06, *p* = 0.041). The main effect of CVD risk was not found to be statistically significant (*b* = 0.34, *p* = 0.072). This finding indicates a stronger increase in overall cognition scores in patients with higher baseline CRP levels, however, with stronger increases in patients with lower degree of CVD risk scores, and in non-smokers.

## Discussion

The main finding of the present study was an inverse relationship at baseline between serum levels of CRP and overall cognitive performance, as well as for the subdomains Delayed memory and Attention, in a clinically relevant sample tested in the acute phase of psychosis. The association was particularly strong for the subgroup with a diagnosis of schizophrenia. At follow-up, which in time correspond to the end of the acute phase, the association was no longer present. The finding was restricted to cognitive performance, as none of the other psychometric parameters tested were associated with the CRP level. The CRP level at baseline predicted the overall cognitive change.

Our results are consistent with those of Dickerson et al. [28], who reported an inverse relationship between CRP levels and cognitive performance but no association towards other measures of psychopathology. Our study adds new knowledge by including a consecutive sample of patients acutely admitted for psychosis, with measurements both at hospital admission and at discharge or after maximally 6 weeks, thus giving the possibility to analyze the data also with regard to longitudinal changes. We argue that this period of time reflects the phase of treatment with the most severe symptoms, supported by the decrease of the CGI-S scores from markedly ill at baseline to mildly affected at follow-up. Interestingly, the negative association between CRP levels and overall cognitive performance was present only at baseline. Since the sample at follow-up was smaller than at baseline, the lack of statistically significant difference at follow-up could be due to insufficient statistical power. However, the correlation was reduced substantially at follow-up, approaching zero, which makes the lack of association unlikely to be a sample size problem. Another possibility might be that the attrition was not at random, giving rise to selection bias at follow-up compared to the sample at baseline. This seems unlikely, however, as attrition was not related to any baseline characteristics. Finally, cognitive dysfunction might theoretically be associated with positive symptoms of psychosis, and the positive symptoms could accordingly confound the association between CRP and cognitive performance, which could also explain why the association between CRP and cognitive performance disappeared in remission. A sensitivity analysis was therefore undertaken to adjust for the potential contribution from positive symptoms, but the results remained unchanged, which is also in line with the finding of a meta-analysis on the subject [65]. Taken together, the inverse association between CRP and cognition may accordingly be interpreted as at least partly state dependent.

In our study, there was no association between CRP or any of the clinical variables, which is not entirely in line with the findings in the meta-analysis by Fernandes et al. [27], who found an association between CRP and positive but not negative symptoms. The meta-analysis was based on about 2,000 schizophrenia subjects and healthy controls, and the apparent lack of an association in our much smaller sample may be related to insufficient statistical power. Our results are, however, in correspondence with the Dickerson et al. [28] study finding no association between CRP and any psychiatric symptoms.

The mean overall cognitive t-score increased significantly between baseline and follow-up, and although the mean CRP levels were almost identical at the assessment points, the distribution changed substantially towards the cognitive t-scores. Furthermore, the baseline CRP level was found to predict change of cognitive performance. Hypotheses regarding the biological substrate mediating the inflammation-related effects on cognition can only be speculative. There is however phase-specific fluctuations reported for both myelin integrity and glutamate levels in schizophrenia [66–70]. Considerable cross-talk has been suggested between immuno-inflammatory processes, myelin, and glutamate in schizophrenia, as recently reviewed by Kroken et al. [21]. CRP is known to activate complement, and the complement system have demonstrated different and somewhat paradoxical effects in the central nervous system (CNS) [71–73]. Some of these effects could be relevant to neurodegeneration and inflammation in several brain disorders including Alzheimer’s disease [72]. Inflammatory processes may also decrease the blood–brain-barrier integrity, leaving the CNS more vulnerable to complement protein infiltration from the periphery [74].

The present study also supplements the findings of Dickerson et al. [28], as more than half the sample was antipsychotic drug naïve, which could be used as a proxy for early stage psychosis. The sub-analyses in the drug-naïve patients revealed similar findings to those of the total sample, with almost identical B and Beta values. The non-significant p-value is probably related to insufficient statistical power in the small sample of drug naïve patients. This interpretation is further supported by the fact that entering medication status into the regression did not change the association between CRP and cognition, which might indicate that the association between CRP levels and cognition was not a result of longstanding psychosis or medication (adverse) effects.

The cognitive deficits in schizophrenia are generally considered to be stable over time [75, 76]. At odds with this, we have previously reported an overall cognitive performance improvement across time that was considered clinically significant, and with some differential cognitive effectiveness among different second generation antipsychotics when data from the whole follow-up period was analyzed [29]. In the present study, all but one patient used antipsychotics at follow-up. It cannot be ruled out that the significant cognitive improvement observed also in the acute phase is antipsychotic drug-related, but due to the lack of a placebo group no firm conclusions can be drawn. It should be noted that anti-inflammatory effects of antipsychotic drugs have been reported in several studies [77, 78], providing a putative link to any cognitive enhancement mediated by the drugs in the present study.

We found significant inverse associations between CRP levels and Delayed memory, and Attention subdomains, respectively, whereas no significant associations were present for the other subdomains. Aas et al. [79] found overall cognitive impairment present already in first episode psychosis patients with the largest effect sizes for verbal memory, executive function, and general IQ. Correspondingly, a global impairment was found across all the subdomains in our sample, the Attention subdomain had the lowest t-score, followed closely by the Immediate memory and Delayed memory subdomains. Seeking reasons for the associations with CRP levels being present only in some of the subdomains is outside the scope of this study. It is nevertheless an interesting observation that the inverse association is present in the subdomains showing the most pronounced impairments which might indicate a greater vulnerability to inflammation-related processes compared to other subdomains of cognition.

Some limitations to the study should be mentioned. Minor elevations in the CRP levels are associated with numerous conditions and although we adjusted for several potential confounders we may have missed other unknown or hidden confounders between CRP levels and cognitive performance. However, most of the conditions associated with minimal CRP elevations do not have an apparent relationship towards cognitive function, making them less likely to confound the results presented here. As already discussed, the attrition rate was substantial, and selection bias cannot be ruled out, although attrition was not related to any baseline characteristics. We used only CRP as a measure of inflammation, and clearly a broader display of inflammatory markers would have added further strength to the study. Finally, although the association found for the primary outcome was highly statistically significant, the explained variance was only modest.

## Conclusions

Despite the limitations mentioned, the data support an inflammatory component to the cognitive impairment in schizophrenia and related psychoses, which may be at least partly state dependent. Several anti-inflammatory agents have shown promising results as add-ons to antipsychotic drug treatment in schizophrenia [80, 81]. Future studies should prospectively and repeatedly examine longitudinal changes in CRP and other markers of inflammation, and their association with cognitive performance. If our results are replicated, anti-inflammatory drugs may be especially beneficial in the acute phase of psychosis for cognitive enhancement.

## References

[1] Tandon R, Nasrallah HA, Keshavan MS (2009). Schizophrenia, “just the facts” 4. Clinical features and conceptualization. Schizophr Res.

[2] Kahn RS, Keefe RS (2013). Schizophrenia is a cognitive illness: time for a change in focus. JAMA psychiatry.

[3] Palmer BW, Dawes SE, Heaton RK (2009). What do we know about neuropsychological aspects of schizophrenia?. Neuropsychol Rev.

[4] Lewandowski KE, Cohen BM, Ongur D (2011). Evolution of neuropsychological dysfunction during the course of schizophrenia and bipolar disorder. Psychol Med.

[5] Keefe RS, Fenton WS (2007). How should DSM-V criteria for schizophrenia include cognitive impairment?. Schizophr Bull.

[6] Green MF (1996). What are the functional consequences of neurocognitive deficits in schizophrenia?. Am J Psychiatry.

[7] Green MF, Kern RS, Braff DL, Mintz J (2000). Neurocognitive deficits and functional outcome in schizophrenia: are we measuring the “right stuff”?. Schizophr Bull.

[8] Kaneda Y, Jayathilak K, Meltzer HY (2009). Determinants of work outcome in schizophrenia and schizoaffective disorder: role of cognitive function. Psychiatry Res.

[9] Laes JR, Sponheim SR (2006). Does cognition predict community function only in schizophrenia?: a study of schizophrenia patients, bipolar affective disorder patients, and community control subjects. Schizophr Res.

[10] Tsang HW, Leung AY, Chung RC, Bell M, Cheung WM (2010). Review on vocational predictors: a systematic review of predictors of vocational outcomes among individuals with schizophrenia: an update since 1998. Aust N Z J Psychiatry.

[11] van Os J, Kapur S (2009). Schizophrenia. Lancet.

[12] Ripke S, O’Dushlaine C, Chambert K, Moran JL, Kahler AK, Akterin S, Bergen SE, Collins AL, Crowley JJ, Fromer M (2013). Genome-wide association analysis identifies 13 new risk loci for schizophrenia. Nat Genet.

[13] Stefansson H, Ophoff RA, Steinberg S, Andreassen OA, Cichon S, Rujescu D, Werge T, Pietilainen OP, Mors O, Mortensen PB (2009). Common variants conferring risk of schizophrenia. Nature.

[14] Schizophrenia Working Group of the Psychiatric Genomics C (2014). Biological insights from 108 schizophrenia-associated genetic loci. Nature.

[15] Havik B, Le Hellard S, Rietschel M, Lybaek H, Djurovic S, Mattheisen M, Muhleisen TW, Degenhardt F, Priebe L, Maier W (2011). The complement control-related genes CSMD1 and CSMD2 associate to schizophrenia. Biol Psychiatry.

[16] Horvath S, Mirnics K (2014). Immune system disturbances in schizophrenia. Biol Psychiatry.

[17] Girgis RR, Kumar SS, Brown AS (2014). The cytokine model of schizophrenia: emerging therapeutic strategies. Biol Psychiatry.

[18] Upthegrove R, Manzanares-Teson N, Barnes NM (2014). Cytokine function in medication-naive first episode psychosis: a systematic review and meta-analysis. Schizophr Res.

[19] Ribeiro-Santos A, Lucio Teixeira A, Salgado JV (2014). Evidence for an immune role on cognition in schizophrenia: a systematic review. Curr Neuropharmacol.

[20] Kahn RS, Sommer IE. The neurobiology and treatment of first-episode schizophrenia. Mol Psychiatry. 2015;20(1):84-97. 10.1038/mp.2014.66 PMC4320288 25048005

[21] Kroken RA, Loberg EM, Dronen T, Gruner R, Hugdahl K, Kompus K, Skrede S, Johnsen E (2014). A critical review of pro-cognitive drug targets in psychosis: Convergence on myelination and inflammation. Frontiers in psychiatry.

[22] Deodhar SD (1989). C-reactive protein: the best laboratory indicator available for monitoring disease activity. Cleve Clin J Med.

[23] Yaffe K, Lindquist K, Penninx BW, Simonsick EM, Pahor M, Kritchevsky S, Launer L, Kuller L, Rubin S, Harris T (2003). Inflammatory markers and cognition in well-functioning African-American and white elders. Neurology.

[24] Ge X, Xu XY, Feng CH, Wang Y, Li YL, Feng B (2013). Relationships among serum C-reactive protein, receptor for advanced glycation products, metabolic dysfunction, and cognitive impairments. BMC Neurol.

[25] Krogh J, Benros ME, Jorgensen MB, Vesterager L, Elfving B, Nordentoft M (2014). The association between depressive symptoms, cognitive function, and inflammation in major depression. Brain Behav Immun.

[26] Dickerson F, Stallings C, Origoni A, Vaughan C, Khushalani S, Yolken R (2013). Elevated C-reactive protein and cognitive deficits in individuals with bipolar disorder. J Affect Disord.

[27] Fernandes BS, Steiner J, Bernstein HG, Dodd S, Pasco JA, Dean OM, Nardin P, Goncalves CA, Berk M. C-reactive protein is increased in schizophrenia but is not altered by antipsychotics: meta-analysis and implications. Mol Psychiatry. 2015. doi:10.1038/mp.2015.87. 10.1038/mp.2015.87 26169974

[28] Dickerson F, Stallings C, Origoni A, Boronow J, Yolken R (2007). C-reactive protein is associated with the severity of cognitive impairment but not of psychiatric symptoms in individuals with schizophrenia. Schizophr Res.

[29] Johnsen E, Jorgensen HA, Kroken RA, Loberg EM: Neurocognitive effectiveness of quetiapine, olanzapine, risperidone, and ziprasidone: A pragmatic, randomized trial. Eur Psychiatry. 2013;28(3):174-84. 10.1016/j.eurpsy.2011.10.003 22153730

[30] Johnsen E, Kroken RA, Wentzel-Larsen T, Jorgensen HA (2010). Effectiveness of second-generation antipsychotics: a naturalistic, randomized comparison of olanzapine, quetiapine, risperidone, and ziprasidone. BMC Psychiatry.

[31] Kay SR, Fiszbein A, Opler LA (1987). The positive and negative syndrome scale (PANSS) for schizophrenia. Schizophr Bull.

[32] Addington D, Addington J, Schissel B (1990). A depression rating scale for schizophrenics. Schizophr Res.

[33] Drake RE, Rosenberg SD, Mueser KT (1996). Assessing substance use disorder in persons with severe mental illness. New Dir Ment Health Serv.

[34] Guy W (1976). Assessment manual for psychopharmacology - revisited, vol. DHHS publ NO ADM 91-338.

[35] Karterud SP G, Loevdahl H, Friis S (1998). Global assessment of functioning - split version (S-GAF): Background and scoring manual.

[36] Beglinger LJ, Gaydos B, Tangphao-Daniels O, Duff K, Kareken DA, Crawford J, Fastenau PS, Siemers ER (2005). Practice effects and the use of alternate forms in serial neuropsychological testing. Arch Clin Neuropsychol.

[37] Gold JM, Queern C, Iannone VN, Buchanan RW (1999). Repeatable battery for the assessment of neuropsychological status as a screening test in schizophrenia I: sensitivity, reliability, and validity. Am J Psychiatry.

[38] Randolph C (1998). RBANS repeatable battery for the assessment of neuropsychological status. Manual.

[39] Goldberg TE, Keefe RS, Goldman RS, Robinson DG, Harvey PD (2010). Circumstances under which practice does not make perfect: a review of the practice effect literature in schizophrenia and its relevance to clinical treatment studies. Neuropsychopharmacology.

[40] Goldberg TE, Goldman RS, Burdick KE, Malhotra AK, Lencz T, Patel RC, Woerner MG, Schooler NR, Kane JM, Robinson DG (2007). Cognitive improvement after treatment with second-generation antipsychotic medications in first-episode schizophrenia: is it a practice effect?. Arch Gen Psychiatry.

[41] Bartels C, Wegrzyn M, Wiedl A, Ackermann V, Ehrenreich H (2010). Practice effects in healthy adults: a longitudinal study on frequent repetitive cognitive testing. BMC Neurosci.

[42] Hausknecht JP, Halpert JA, Di Paolo NT, Moriarty Gerrard MO (2007). Retesting in selection: a meta-analysis of coaching and practice effects for tests of cognitive ability. The Journal of applied psychology.

[43] Wilk CM, Gold JM, Bartko JJ, Dickerson F, Fenton WS, Knable M, Randolph C, Buchanan RW (2002). Test-retest stability of the Repeatable Battery for the Assessment of Neuropsychological Status in schizophrenia. Am J Psychiatry.

[44] Randolph C, Tierney MC, Mohr E, Chase TN (1998). The Repeatable Battery for the Assessment of Neuropsychological Status (RBANS): preliminary clinical validity. J Clin Exp Neuropsychol.

[45] Singh B, Chaudhuri TK (2014). Role of C-reactive protein in schizophrenia: An overview. Psychiatry Res.

[46] Meltzer HY. Update on Typical and Atypical Antipsychotic Drugs. Annu Rev Med. 2013;64:393-406. 10.1146/annurev-med-050911-161504 23020880

[47] Dieset I, Hope S, Ueland T, Bjella T, Agartz I, Melle I, Aukrust P, Rossberg JI, Andreassen OA (2012). Cardiovascular risk factors during second generation antipsychotic treatment are associated with increased C-reactive protein. Schizophr Res.

[48] O’Loughlin J, Lambert M, Karp I, McGrath J, Gray-Donald K, Barnett TA, Delvin EE, Levy E, Paradis G (2008). Association between cigarette smoking and C-reactive protein in a representative, population-based sample of adolescents. Nicotine Tob Res.

[49] Dietrich T, Garcia RI, de Pablo P, Schulze PC, Hoffmann K (2007). The effects of cigarette smoking on C-reactive protein concentrations in men and women and its modification by exogenous oral hormones in women. Eur J Cardiovasc Prev Rehabil.

[50] Sacco KA, Termine A, Seyal A, Dudas MM, Vessicchio JC, Krishnan-Sarin S, Jatlow PI, Wexler BE, George TP (2005). Effects of cigarette smoking on spatial working memory and attentional deficits in schizophrenia: involvement of nicotinic receptor mechanisms. Arch Gen Psychiatry.

[51] Heishman SJ, Kleykamp BA, Singleton EG (2010). Meta-analysis of the acute effects of nicotine and smoking on human performance. Psychopharmacology (Berl).

[52] Ghazavi A, Mosayebi G, Solhi H, Rafiei M, Moazzeni SM (2013). Serum markers of inflammation and oxidative stress in chronic opium (Taryak) smokers. Immunol Lett.

[53] Costello EJ, Copeland WE, Shanahan L, Worthman CM, Angold A (2013). C-reactive protein and substance use disorders in adolescence and early adulthood: a prospective analysis. Drug Alcohol Depend.

[54] Ridker PM (2007). Inflammatory biomarkers and risks of myocardial infarction, stroke, diabetes, and total mortality: implications for longevity. Nutr Rev.

[55] Ridker PM (2007). C-reactive protein and the prediction of cardiovascular events among those at intermediate risk: moving an inflammatory hypothesis toward consensus. J Am Coll Cardiol.

[56] Yaffe K, Kanaya A, Lindquist K, Simonsick EM, Harris T, Shorr RI, Tylavsky FA, Newman AB (2004). The metabolic syndrome, inflammation, and risk of cognitive decline. JAMA.

[57] Yaffe K, Weston AL, Blackwell T, Krueger KA (2009). The metabolic syndrome and development of cognitive impairment among older women. Arch Neurol.

[58] Muthén LK, Muthén BO (2014). Mplus 7.2.

[59] Stoolmiller M, Gottman JM, Collins JM (1995). Using latent growth curve models to study developmental processes. The analysis of change.

[60] Muthén LK, Muthén BO (2008). Mplus 5.2.

[61] Kline RB (2010). Principles and practice of structural equation modeling.

[62] Duncan TE, Duncan SC (2004). An introduction to latent growth curve modeling. Behav Ther.

[63] Bollen KA, Curran PJ (2006). Latent curve models: A structural equation perspective.

[64] Cohen J, Cohen P, West SG, Aiken LS (2003). Applied multiple regression - correlation analysis for the behavioral sciences.

[65] Dominguez Mde G, Viechtbauer W, Simons CJ, van Os J, Krabbendam L (2009). Are psychotic psychopathology and neurocognition orthogonal? A systematic review of their associations. Psychol Bull.

[66] Garver DL, Holcomb JA, Christensen JD (2008). Compromised myelin integrity during psychosis with repair during remission in drug-responding schizophrenia. Int J Neuropsychopharmacol.

[67] Theberge J, Bartha R, Drost DJ, Menon RS, Malla A, Takhar J, Neufeld RW, Rogers J, Pavlosky W, Schaefer B (2002). Glutamate and glutamine measured with 4.0 T proton MRS in never-treated patients with schizophrenia and healthy volunteers. Am J Psychiatry.

[68] Ota M, Ishikawa M, Sato N, Hori H, Sasayama D, Hattori K, Teraishi T, Nakata Y, Kunugi H (2012). Glutamatergic changes in the cerebral white matter associated with schizophrenic exacerbation. Acta Psychiatr Scand.

[69] Ohrmann P, Siegmund A, Suslow T, Spitzberg K, Kersting A, Arolt V, Heindel W, Pfleiderer B (2005). Evidence for glutamatergic neuronal dysfunction in the prefrontal cortex in chronic but not in first-episode patients with schizophrenia: a proton magnetic resonance spectroscopy study. Schizophr Res.

[70] Egerton A, Brugger S, Raffin M, Barker GJ, Lythgoe DJ, McGuire PK, Stone JM (2012). Anterior cingulate glutamate levels related to clinical status following treatment in first-episode schizophrenia. Neuropsychopharmacology.

[71] Mold C, Gewurz H, Du Clos TW (1999). Regulation of complement activation by C-reactive protein. Immunopharmacology.

[72] Bonifati DM, Kishore U (2007). Role of complement in neurodegeneration and neuroinflammation. Mol Immunol.

[73] Sjoberg AP, Trouw LA, Blom AM (2009). Complement activation and inhibition: a delicate balance. Trends Immunol.

[74] Jacob A, Alexander JJ (2014). Complement and blood-brain barrier integrity. Mol Immunol.

[75] Nuechterlein KH, Ventura J, Subotnik KL, Bartzokis G (2014). The early longitudinal course of cognitive deficits in schizophrenia. J Clin Psychiatry.

[76] Keefe RS (2014). The longitudinal course of cognitive impairment in schizophrenia: an examination of data from premorbid through posttreatment phases of illness. J Clin Psychiatry.

[77] Meyer U, Schwarz MJ, Muller N (2011). Inflammatory processes in schizophrenia: a promising neuroimmunological target for the treatment of negative/cognitive symptoms and beyond. Pharmacol Ther.

[78] Monji A, Kato TA, Mizoguchi Y, Horikawa H, Seki Y, Kasai M, Yamauchi Y, Yamada S, Kanba S (2013). Neuroinflammation in schizophrenia especially focused on the role of microglia. Prog Neuropsychopharmacol Biol Psychiatry.

[79] Aas M, Dazzan P, Mondelli V, Melle I, Murray RM, Pariante CM (2014). A systematic review of cognitive function in first-episode psychosis, including a discussion on childhood trauma, stress, and inflammation. Frontiers in psychiatry.

[80] Miyamoto S, Miyake N, Jarskog LF, Fleischhacker WW, Lieberman JA (2012). Pharmacological treatment of schizophrenia: a critical review of the pharmacology and clinical effects of current and future therapeutic agents. Mol Psychiatry.

[81] Sommer IE, van Westrhenen R, Begemann MJ, de Witte LD, Leucht S, Kahn RS (2014). Efficacy of anti-inflammatory agents to improve symptoms in patients with schizophrenia: an update. Schizophr Bull.

